# Synergistic Solvation as the Enhancement of Local Mixing

**DOI:** 10.1021/acs.jpcb.4c01582

**Published:** 2024-06-03

**Authors:** Seishi Shimizu, Nobuyuki Matubayasi

**Affiliations:** † York Structural Biology Laboratory, Department of Chemistry, University of York, Heslington, York YO10 5DD, United Kingdom; ‡ Division of Chemical Engineering, Graduate School of Engineering Science, Osaka University, Toyonaka, Osaka 560-8531, Japan

## Abstract

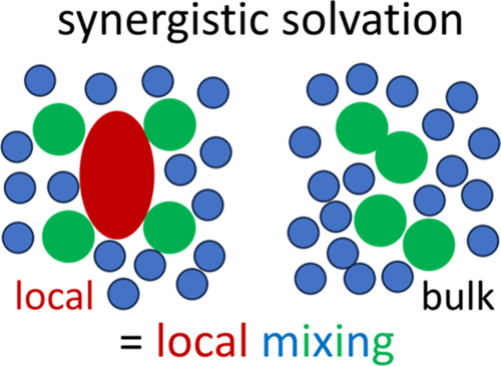

Mixing two solvents can sometimes make a much better solvent than expected from their weighted mean. This phenomenon, called synergistic solvation, has commonly been explained via the Hildebrand and Hansen solubility parameters, yet their inability in other solubilization phenomena, most notably hydrotropy, necessitates an alternative route to elucidating solubilization. While, recently, the universal theory of solubilization was founded on the statistical thermodynamic fluctuation theory (as a generalization of the Kirkwood–Buff theory), its demand for experimental data processing has been a hindrance for its wider application. This can be overcome by the solubility isotherm theory, which is founded on the fluctuation theory yet reduces experimental data processing significantly to the level of isotherm analysis in sorption. The isotherm analysis identifies the driving force of synergistic solvation as the enhancement of solvent mixing around the solute, opposite in behavior to hydrotropy (characterized by the enhancement of demixing or self-association around the solute). Thus, the fluctuation theory, including its solubility isotherms, provides a universal language for solubilization across the historic subcategorization of solubilizers, for which different (and often contradictory) mechanistic models have been proposed.

## 1 Introduction

**Figure 1:**
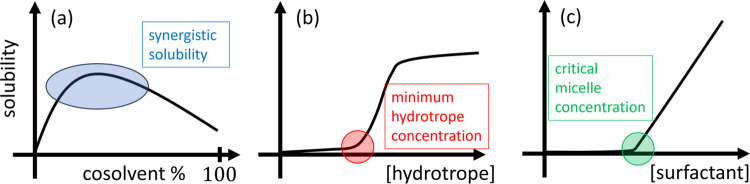
Schematic diagram showing the solubility isotherms in the presence of (a) cosolvents, (b) hydrotropes, and (c) surfactants. (a) Synergistic solubility is the existence of a solubility peak above the solubilities in pure solvent and cosolvent. (b) Minimum hydrotrope concentration is a sudden onset of solubility increase, typically around 0.5 M. (c) Around CMC, a sudden onset of solubilization is observed, above which solubility increases linearly.

Low solubility is a major hindrance to formulation processes.^1^ This can be overcome, however, by adding solubilizer molecules. The “solubilizer” is a general terminology adopted in this paper, which encompasses different subcategories, commonly referred to as (a) cosolvents, which can mix with solvents at high concentrations (Figure 1a),^2−4^ as well as (b) hydrotropes (Figure 1b)^5−7^ and (c) surfactants (Figure 1c)^7−14^ that are usually added in dilution to water.

Our goal is to establish a universal theory of solubilization. So far, we have clarified how hydrotropes^1,7^ and surfactants^15^ work, based on the statistical thermodynamic fluctuation theory, whose applicability ranges from solutions,^16−18^ macromolecules and colloids,^1,19^ and interfaces.^20^ However, an important class of solubilization phenomena, synergistic solvation, remains to be elucidated beyond the current limitations, as summarized below.

### 1.1 Synergistic Solvation

When the solubility in a binary solvent mixture is higher than expected from those in pure solvents (shown schematically in Figure 1a), synergistic solvation takes place. Its mechanism, according to the regular solution model,^21^ is the matching of solubility parameters: the solubility parameter for the mixed solvent (calculated via weighted averaging of the pure solvent values) matches that of the solute.^22−24^ This approach was adopted later by the three-dimensional solubility parameters by Hansen.^24^ However, such an approach is not only dependent on a series of model assumptions (as will be made clear in Section 3) but also limited only to the positive deviation from ideality.^25^

This necessitates a renewed quest for understanding the mechanism of synergistic solvation on a molecular basis. To do so, the key is the solubility isotherm (i.e., a plot of solubility against solubilizer concentration, Figure 1), whose shape contains information on the underlying solubility mechanism.^1,7,15^ In the following, we will survey the two modern theoretical tools available for elucidating solubility isotherms.

### 1.2 Kirkwood–Buff Theory

Hydrotropes are a loosely defined class of solubilizers, most commonly small molecules with weak amphiphilicity, which do not exhibit critical micelle concentrations (CMC).^1,5,6^ Classically, hydrotrope preaggregation (i.e., self-association in the bulk) was considered to be the driving force for solubilization.^6,26^ Despite the lack of micelle formation, an analogy between the threshold hydrotrope concentration for solubilization (i.e., the minimum hydrotrope concentration) and micellar solubilization has been invoked by some authors.^27,28^ However, the Kirkwood–Buff (KB) theory of solutions,^16−19,29^ an exact, model-free theory from classical statistical thermodynamics, has shown that the hydrotrope self-association in the bulk decreases solubilization efficiency,^30−32^ contrary to the classical hypothesis.

Before the application of the KB theory, it was necessary to classify solubilizers into subcategories (such as hydrotropes, cosolvents, and surfactants) and to develop a different model for each.^30−32^ The KB theory was game-changing in its ability to quantify the interactions between every pair of species in the solution, based directly on experimental data,^16−19,29^ to identify the interactions that influence solubilization dominantly and to achieve the above quantification and identification without introducing any model assumptions.

#### Need for Simplification

The rigorous nature of the KB theory has also been the source of difficulty, which can be appreciated by considering solubility in a binary mixture. For this system, six KB integrals (KBIs; solute–solvent, solute–solubilizer, solvent–solvent, solubilizer–solubilizer, solubilizer–solvent and solute–solute) need to be evaluated, requiring 6 different sets of thermodynamic data as an input; not only solubility but also density, activity coefficients, and compressibility measured extensively as a function of composition. Focusing on dilute solutes reduces the number of KBIs only by one.^32^ Because of such difficulty, a full determination of KBIs in concentrated ternary solutions has rarely been performed.^33−37^ However, only a few KBIs (such as the solute–solubilizer and solubilizer–solubilizer) turned out to be the key to understanding solubilization.^15,32^ This implies that the solubilization mechanism could be revealed via a simpler route. Such a simplification will be carried out in this work.

### 1.3 Cooperative Solubilization

Understanding the origin of the abrupt solubilization onset at the “minimum hydrotrope concentration”^27,28^ necessitated us to go beyond the KB theory. We have developed a theory of cooperative solubilization,^30−32^ which was successful in attributing the onset of solubilization to the enhancement of hydrotrope self-association around the solute. This has led to replacing the classical hydrotrope preaggregation hypothesis with the cooperative hydrotrope association around the solute.^30−32^ This conclusion applies not only to hydrotropes alone but also to surfactants, for which the sudden onset of solubilization comes from the enhanced surfactant aggregation around CMC.^15^

#### Need for Simplification

The application of the cooperative solubilization theory, despite its universality, has been limited to the onset of solubilization (such as the minimum hydrotrope concentration and CMC) because of its theoretical complexity. To overcome this limitation, we have developed the cooperative solubility isotherm for hydrotropes,^38^ expressed via a simple analytical equation for capturing solute-induced hydrotrope association, which can be used to fit experimental solubility data.^30−32^ However, its success has been limited to hydrotropes.^39,40^ A solubility isotherm based on the statistical thermodynamic fluctuation theory, which can be applicable to synergistic solubility, is not available until now.

### 1.4 Need for a Solubility Isotherm

Our goal is to reveal the mechanisms of solubilization via solubility isotherms as a novel, simpler alternative to the KB and cooperative solubilization theories that are exact yet complicated for applications.^38^ To achieve this goal, important lessons come from sorption isotherms. First, the recently established analogy between solvation and sorption enables the application of the theoretical tools for sorption to solubilization, across their difference in the thermodynamic degrees of freedom.^19,41,42^ Second, our recent sorption isotherms enable a statistical thermodynamic interpretation (such as number fluctuations and KBIs) of experimental data through only a few parameters.^43−45^ Such an approach is less demanding in data acquisition and processing than the KB theory.^20,43,46,47^ Third, cumbersome thermodynamic variable transformation, indispensable for converting experimental data to KBIs, has been made more efficient by statistical variable transformation.^48,49^

### 1.5 Our Aims

Armed with the modern theoretical tools summarized above, we will implement our aims (see the opening paragraph) with the following objectives:

a. To establish an isotherm approach to elucidating solubilization mechanisms as a facile alternative to the KB theory.
b. To derive the polynomial solubility isotherm to capture solute–solubilizer preferential interaction and the enhancement or reduction of self-association around the solute.
c. To show that synergistic solvation and cooperative solubilization have the opposite behavior in terms of the solute’s role in solubilizer self-association.

To quantify the self-association of solubilizers and solvents, we will employ the Kirkwood–Buff χ (KB χ) parameter, which we introduced recently as the generalization of the Flory χ and employed to elucidate sorption.^43,44^

## 2 Theoretical Methods

### 2.1 Fluctuation Theory

Consider the solubility of a solute (denoted as species *u*) in a mixture consisting of a solvent (species 1) and a solubilizer (species 2). (Note that the terms “solvent” and “solubilizer” have been introduced to facilitate comparison across different classes of solubilization. For synergistic solvation, the “solubilizer” simply refers to the component whose concentration is increased when plotting the solubility isotherm.) Let *N*_*i*_ be the number of species *i* molecules, ⟨*N*_*i*_⟩ be its ensemble average, and δ*N*_*i*_ = *N*_*i*_ – ⟨*N*_*i*_⟩ be its deviation from the mean. Our starting point is the fundamental relationship from the fluctuation solution theory on how the solvation free energy of a solute (μ_u_^*^, i.e., pseudochemical potential) depends on the chemical potential of the solubilizer (μ_2_),
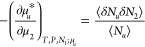
1awhich is linked to the solute–solubilizer number correlation. (For the derivation of eq 1a, see eq 35 of ref (48) with the indexes 1 and 2 swapped.) Note that eq 1a, derived under phase equilibrium between the solute in its pure phase and in solution (constant μ*_u_* in equilibrium with the pure solute phase), is valid for any solute concentration.^15^ (This means that the equation of the same form applies to solutes with sparse solubility, including the examples analyzed in this paper.) Here, for mathematical simplicity, we adopt a {*T*, *P*, *N*_1_, μ_2_, μ*_u_*} ensemble,^50,51^ yet converting to the grand canonical ensemble {*T*, *V*, μ_1_, μ_2_, μ*_u_*}, commonly adopted for the KB theory,^16−18^ is straightforward via statistical variable transformation (Appendix A).^48,49^

Here, we rewrite eq 1a in the format suitable for solubility isotherms. This can be achieved using the well-known relationships, first between the solvation free energy and solubility *c*_u_, dμ_u_^*^ = −*RT*d ln *c_u_*,^52^ which can be derived straightforwardly from the basic relationship between μ_u_ and μ_u_^*^ (see Appendix A), and second between the chemical potential and solubilizer activity, dμ_2_ = *RT*d ln *a*_2_, through which we obtain
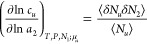
1bSee Appendix A for its derivation.

Improved clarity can be attained in the application of eq 1b by introducing an inhomogeneous ensemble, which contains a fixed solute molecule at the origin as the source of an external field for the solution mixture.^53,54^ The ensemble average in the inhomogeneous ensemble, ⟨⟩*_u_*, is the conditional mean in the presence of a fixed solute molecule, which is related to ⟨⟩ (i.e., the “homogeneous” mean), via
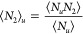
2Through eq 2, eq 1b can be expressed in the following simple form
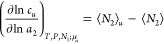
3Equation 3, being a rigorous relationship, is the theoretical foundation for solubility isotherms that will be derived in this paper.

### 2.2 Solubility Isotherms

Our aim is not only to derive solubility isotherm equations for fitting experimental data but also to quantify the interactions underlying solubilization through the fitting parameters. Just like sorption isotherms,^43−45^ there may be multiple isotherm equations serving different subclasses of solubilization. Indeed, our previous theory of cooperative solubilization^38^ is in fact a solubility isotherm for hydrotropes that exhibit a sigmoidal functional shape. In this paper, we will derive the polynomial isotherm, founded on the *a*_2_-dependence of ⟨*N*_2_⟩*_u_* – ⟨*N*_2_⟩ and thereafter employ *x*_2_ (solubilizer mole fraction) as the variable, to conform to the experimental practice. The polynomial isotherms will reveal the molecular interactions underlying nonlinear solubilization (Section 3) and will serve as facile alternatives to the KB theory.^19,29,30,32^

#### 2.2.1 Polynomial Isotherm

Here, we derive the polynomial isotherm by expanding ⟨*N*_2_⟩*_u_* – ⟨*N*_2_⟩ in terms of solubilizer activity, *a*_2_, as
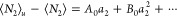
4awhere the parameters *A*_0_ and *B*_0_ are defined at the *a*_2_ → 0 limit as
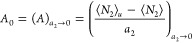
4b
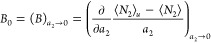
4cwhere the subscript 0 is the shorthand for *a*_2_ → 0, which will be used throughout this paper. Note that the lowest-order term in eq 4a is *A*_0_*a*_2_ because ⟨*N*_2_⟩ – ⟨*N*_2_⟩*_u_* tends to 0 as *a*_2_ → 0. Combining eqs 3 and 4a yields
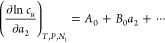
5aand integrating eq 5a (Appendix B) yields
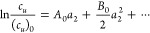
5bwhere (*c_u_*)_0_ is the molar solubility in the absence of the solubilizer at *a*_2_ = 0. Equation 5b will be referred to as the polynomial isotherm.

In the above, the polynomial isotherm was truncated at the second order of *a*_2_, and the second-order expansion with respect to the mole fraction of the solubilizer *x*_2_ will be discussed in Section 3.2.2. Synergistic solvation is typically expressed in the form of quadratic dependence of the solubility on *a*_2_ or *x*_2_, in which case eq 5b is enough to capture the physical meanings of the coefficients involved in the quadratic form. If a quadratic fit is insufficient, our formulation can be straightforwardly extended to incorporate higher-order terms beyond the quadratic in eq 5b.

#### 2.2.2 Interpreting *A*_0_ and *B*_0_

The fluctuation theory provides an interpretation of the isotherm parameters, *A*_0_ and *B*_0_. Statistical variable transformation^48,49^ enables a straightforward conversion between different ensembles (see Appendices B and C), through which *A*_0_, defined via eq 4b in the {*T*, *P*, *N*_1_, μ_2_, μ*_u_*} ensemble,^50,51^ can be expressed in the grand canonical ({*T*, *V*, μ_1_, μ_2_, μ*_u_*}) ensemble as
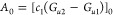
6awhere *c*_1_ is the mole per volume of the pure solvent, *V* is the volume of the system, and *G*_*u*2_ and *G*_*u*1_ are the solute–solubilizer and solute–solvent KBIs. The subscript 0 expresses the *a*_2_ → 0 limit. The parameters for the polynomial solubilization isotherm are defined and evaluated at this limit, just like the ones for the statistical thermodynamic sorption isotherms.^43,44,46,55^ As is clear from eq 6a, *A*_0_ is the difference between the solute–solubilizer and solute–solvent KBIs, commonly referred to as the preferential interaction of solubilizers.^50,51^

Likewise, *B*_0_, defined via eq 4c in the {*T*, *P*, *N*_1_, μ_2_, μ*_u_*} ensemble, can be transformed into the grand canonical ensemble {*T*, *V*, μ_1_, μ_2_, μ*_u_*} as (Appendix C)
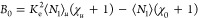
6bwhere *K*_e_ is the equilibrium constant for swapping “a solvent at the vicinity of the solute” with “a solubilizer in the bulk,” and χ*_u_* and χ_0_, the KB χ parameters around the solute and in the bulk,^44^ are defined as
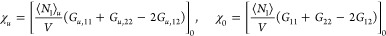
6cχ_u_ and χ_0_ signify the net self-interaction (solvent–solvent and solubilizer–solubilizer, as compared to solubilizer–solvent) around the solute and in the bulk, respectively. Both χ*_u_* and χ_0_ have been defined at the *a*_2_ → 0 limit. The KB χ (eq 6c) was introduced as the generalization of the Flory χ.^25,44^ While the Flory χ has been derived in the framework of the lattice model of solutions under mean-field approximation, the KB χ parameter is model- and approximation-free. Using the KB χ, *B*_0_ can be interpreted as the solute-induced enhancement of the self-interaction. (Note that *B*_0_/*A*_0_ will later be employed for fitting and interpretation, which will be shown shortly to be free of ⟨*N*_1_⟩*_u_* and ⟨*N*_1_⟩ as found in eq 6b, which sharpens its character as the extent of the solute-induced enhancement of the self-interaction).

The KB χ for the bulk solution (eq 6c) appears also in the conversion of *a*_2_ to the mole fraction *x*_2_ (see Appendix C), via
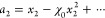
6dwhich shows that the KB χ captures the nonideality of binary solution mixtures in a manner analogous to the Flory χ.^25^ However, the common adoption of the mixing rule (especially in the solubility parameters) has prevented the Flory χ-based approaches from recognizing the importance of the solute-induced enhancement or reduction of self-interactions (eq 6c).

Combining eqs 5b and 6d, we obtain the following logarithmic solubility isotherm as a function of *x*_2_
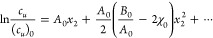
7awhich can also be expressed in the (linear-)solubility representation as
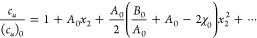
7bThe parameters *A*_0_ and χ_0_ have already given interpretations (eqs 6a and 6c). Combining eqs 6a and 6b,  can be expressed (Appendices C and D) as
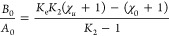
7cwhere *K*_2_ is the bulk–solute vicinity partition coefficient of the solubilizer. Based on eq 7c,  is interpreted as the solute-induced enhancement of self-association (from χ_0_ in the bulk to χ*_u_* around the solute).

Thus, the two representations of the polynomial isotherm, ln *c_u_*/(*c_u_*)_0_ and *c_u_*/(*c_u_*)_0_, will both be useful in the analysis and interpretation of experimental isotherms, as will be discussed in Section 3. The polynomial solubilization isotherm bears similarity to the polynomial sorption isotherm in its parameters and interpretation.^44^ To summarize, we have established the polynomial solubility isotherm (eqs 7a and 7b), whose parameters contain contributions from the preferential solute–solubilizer interaction (*A*_0_), the enhancement of self-interaction around the solute (*B*_0_/*A*_0_), and the bulk χ.

#### 2.2.3 Significance of the Polynomial Isotherm

The polynomial solubility isotherm, in its two representations (eqs 7a and 7b), is founded on the *a*_2_-expansion of solubilization (eq 5b) and the *x*_2_-expansion of *a*_2_ (eq 6d). While these expansions are exact, truncating the expansion leads to approximation. The parameters of the expansions (eqs 5b and 6d), as well as of the polynomial solubility isotherm (eqs 7a and 7b), are defined at the *x*_2_ → 0 limit. This is not an approximation but is a logical consequence of the Maclaurin expansions (eqs 5b and 6d) underlying the solubility isotherm. In this way, our theory can be considered as the generalization of the McMillan–Mayer theory of osmotic pressure, which involves a series expansion of the osmotic pressure in terms of the solute concentration.^56^ Its parameters (i.e., the virial coefficients) are defined at the zero solute concentration, analogous to our polynomial solubility isotherm. Approximation can be made via the truncation of the expansion, whose sufficiency can be informed through fitting experimental data.

The novelty of the solubility isotherm is threefold. First, in contrast to the conventional KBI calculations at each concentration,^30^ the solubility isotherm synthesizes the predictive nature of an isotherm equation and the capability for a rigorous molecular interpretation via its parameters. Note that the concept of solute-induced self-association (which is contained in *B*_0_) was beyond the reach of the traditional KBIs (which are, by definition, binary). This novel concept was introduced by us initially via the inhomogeneous solution theory,^14^ thereafter through the cooperative solubilization model applicable for hydrotropes at low concentration,^38^ and finally as one of the characteristic parameters for the sorption isotherms.^44^ Second, how the solute-induced self-association affects the shape of the solubility isotherm over a wide composition range has now been clarified, which was not possible with our previous approach.^14^ Third, our solubility isotherm constitutes a part of a universal approach spanning from solution to sorption, founded on the mathematical analogy between solvation and sorption, which has only recently been formulated rigorously.^19,41,42^

## 3 Results and Discussion

### 3.1 Synergistic Solvation

#### 3.1.1 Synergy via Quadratic Isotherm

Our goal is to reveal the mechanism of synergistic solvation and clarify how it differs from hydrotropy. Our theoretical foundation is the polynomial isotherm derived from the fluctuation solution theory (eqs 7a and 7b). The strict definition of synergistic solvation has not been established, yet “a binary solvent mixture exhibits a higher solubility than either of the component solvents alone” may be the most general one at this stage.^4^ Note that synergistic solvation is “also known as parabolic solubility”^4^ due to the ubiquitousness of the parabolic solubility isotherms.^3,57,58^ Hence, the present paper focuses on the quadratic isotherm as the simplest and most common class of synergistic solvation as the first step. (For the solubility isotherms with more complex functional shapes, cubic or higher-order terms in *x*_2_ may be incorporated.)

#### 3.1.2 Logarithmic versus Linear Plots of Solubility

**Figure 2:**
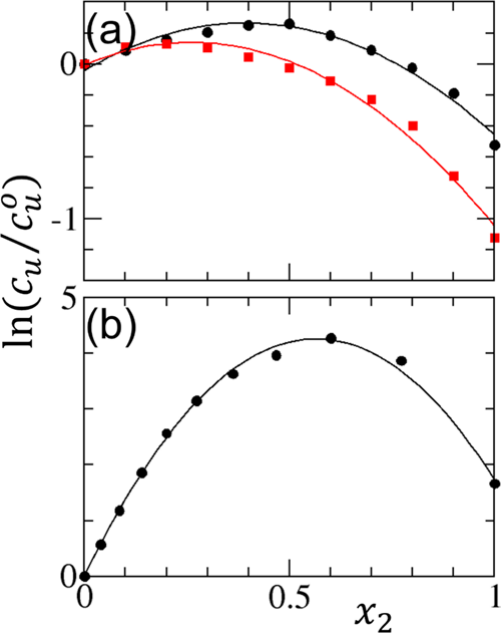
Application of the quadratic solubility isotherm (eq 8) to fit the logarithmic scale solubility data (ln *c*_*u*_/(*c*_*u*_)_0_), plotted against the mole fraction *x*_2_. (a) Solubility of benzoin (species *u*) in the mixtures of ethyl acetate (species 1) and alcohol (species 2) at 298.15 K (black circles for methanol and red squares for ethanol), based on the data reported by Yang et al.,^57^ in combination with the density data of alcohol–ethyl acetate mixtures published by Nikam et al.^59^ (b) Solubility of rivaroxaban (species *u*) in the mixture of methanol (species 1) and dichloromethane (species 2) at 293.15 K, based on the data reported by Jeong et al.,^58^ in combination with the density data by Damyanov and Velchev.^60^ The fitting parameters are summarized in Table 1.

**Figure 3:**
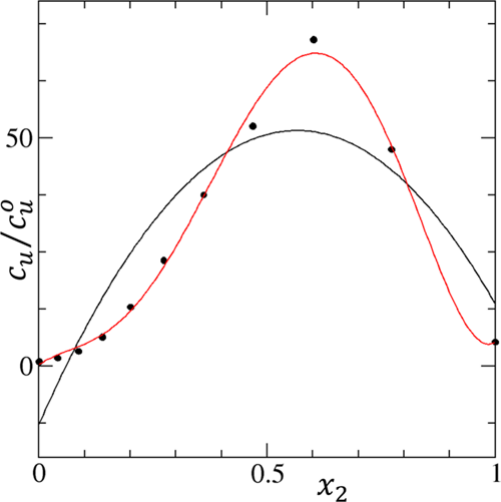
Linear solubility isotherm, *c*_*u*_/(*c*_*u*_)_0_ against *x*_2_, of rivaroxaban (species *u*) in the mixture of methanol (species 1) and dichloromethane (species 2), to be compared with the logarithmic isotherm in Figure 2b. A quadratic isotherm (*c*_*u*_/(*c*_*u*_)_0_ = −12.969 + 228.27*x*_2_ – 201.65*x*_2_^2^, black line) was insufficient to fit the experimental data (black circles), and the fifth-order polynomial was required for a reasonable fit (red line; = 0.2061 + 70.076*x*_2_ – 523.91*x*_2_^2^ + 3304.7*x*_2_^3^ – 5355.1*x*_2_^4^ + 2509.2*x*_2_^5^). This complication contrasts with the ease of fitting by the logarithmic isotherm (Figure 2b).

Here, we show that the logarithmic representation (eq 7a) of the polynomial isotherm is superior to the linear representation (eq 7b) in fitting experimental solubility data. To demonstrate this, we have chosen the solubility of benzoin in ethyl acetate–methanol and ethyl acetate–ethanol mixtures^57,59^ and the solubility of rivaroxaban in methanol–dichloromethane mixtures.^58,60^ While the logarithmic representation leads to an excellent fit incorporating up to *x*_2_^2^ (Figure 2), the linear representation exhibited fitting difficulties, requiring higher-order terms (Figure 3). Note that a minor adjustment was involved for the logarithmic representation (eq 7a) with an adjustable constant, ϵ, as
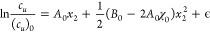
8leading to a good fit over the entire range of *x*_2_ with a negligibly small ϵ (see Appendix B for its necessity). The fitting parameters are summarized in Table 1, from which the interaction parameters have been calculated (Table 2). Although the linear representation has been adopted widely to report experimental solubility isotherms, we advocate for a logarithmic representation based not only on the facility for fitting but also on its directness in interpreting the fitting parameters.

**Table 1:** Fitting Parameters for the Logarithmic Representation of the Quadratic Solubility Isotherm (Equation 8)

solute	solvent	cosolvent	*A*_0_	*B*_0_ – 2*A*_0_χ_0_	ϵ
benzoin	ethyl acetate	methanol	1.56	–3.93	–0.044
benzoin	ethyl acetate	ethanol	1.14	–4.34	–0.011
rivaroxaban	methanol	dichloromethane	15.1	–26.6	–0.0036

**Table 2:** Interaction Parameters Evaluated Based on Table 1

solute	solvent	cosolvent	χ_0_	*A*_0_	*B*_0_	
benzoin	ethyl acetate	methanol	1.91^a^	1.56	2.03	–2.52
benzoin	ethyl acetate	ethanol	1.32^a^	1.14	–1.33	–3.80
rivaroxaban	methanol	dichloromethane	1.06^a^	15.1	5.42	–1.76

a Calculated from Table 13–4 of ref (71) and ref (72) which can be transformed to χ_0_ = 2α via eq C8.

#### 3.1.3 Conditions for Synergy

According to its definition, higher solubility in the mixture is observed for synergistic solvation than is expected from the weighted averaging of solubilities in pure solvents. Based on the logarithmic representation of the polynomial isotherm (eq 7a), here we clarify the mechanism underlying the solubility maximum. As a first step, we rewrite eq 7a as
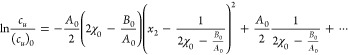
9For eq 9 to exhibit a maximum,  must be positive. (This is in line with the negative  in Table 2). Under this condition, the maximum solubility, *c*_*u*_^max^, is expressed as
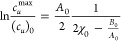
10aFor *c*_*u*_^max^ to be greater than (*c_u_*)_0_,  must be positive. This condition is equivalent to the above positiveness condition for . In addition, the maximum solubility at
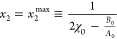
10bmust be located between *x*_2_ = 0 and 1. *x*_2_^max^ > 0 leads to . Consequently, *A*_0_ > 0 results in combination with the maximum and synergy conditions. The other end, *x*_2_^max^ < 1, leads to . Taking all together, we can summarize the conditions following for synergistic solubilization as
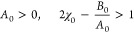
10cwhich is indeed satisfied by all of the examples (Table 2).

#### 3.1.4 Mechanism of Synergy

Having identified the range of parameters that exhibit synergistic solvation (eq 10c), here we clarify its significance on a mechanistic basis. To do so, let us start by summarizing the interpretations of the parameters involved:

a. *A*_0_ for preferential solute–solubilizer interaction (eq 6a)
b. for the self-association (KB χ) enhancement around the solute (eq 7c)
c. χ_0_ for bulk-phase self-association (eq 6c).

The interpretation of the parameters (a–c) will now reveal the mechanism of synergistic solubilization (eq 10c). First, *A*_0_ > 0 in eq 10c signifies preferential solute–solubilizer interaction compared to solute–solvent. Second,  in eq 10c signifies sufficiently stronger bulk-phase self-association (χ_0_) compared to the self-association enhancement around the solute (*B*_0_/*A*_0_). This can be rephrased more intuitively by rewriting it as . Now,  signifies the enhancement of solvent–solubilizer mixing (self-dissociation), whereas −χ_0_ means the solvent–solubilizer mixing in the bulk. The rephrased inequality shows that mixing, enhanced around the solute, is stronger than the bulk-phase mixing of solvent and solubilizer.

#### 3.1.5 Synergy versus Cooperative Solubilization by Hydrotropes

**Figure 4:**
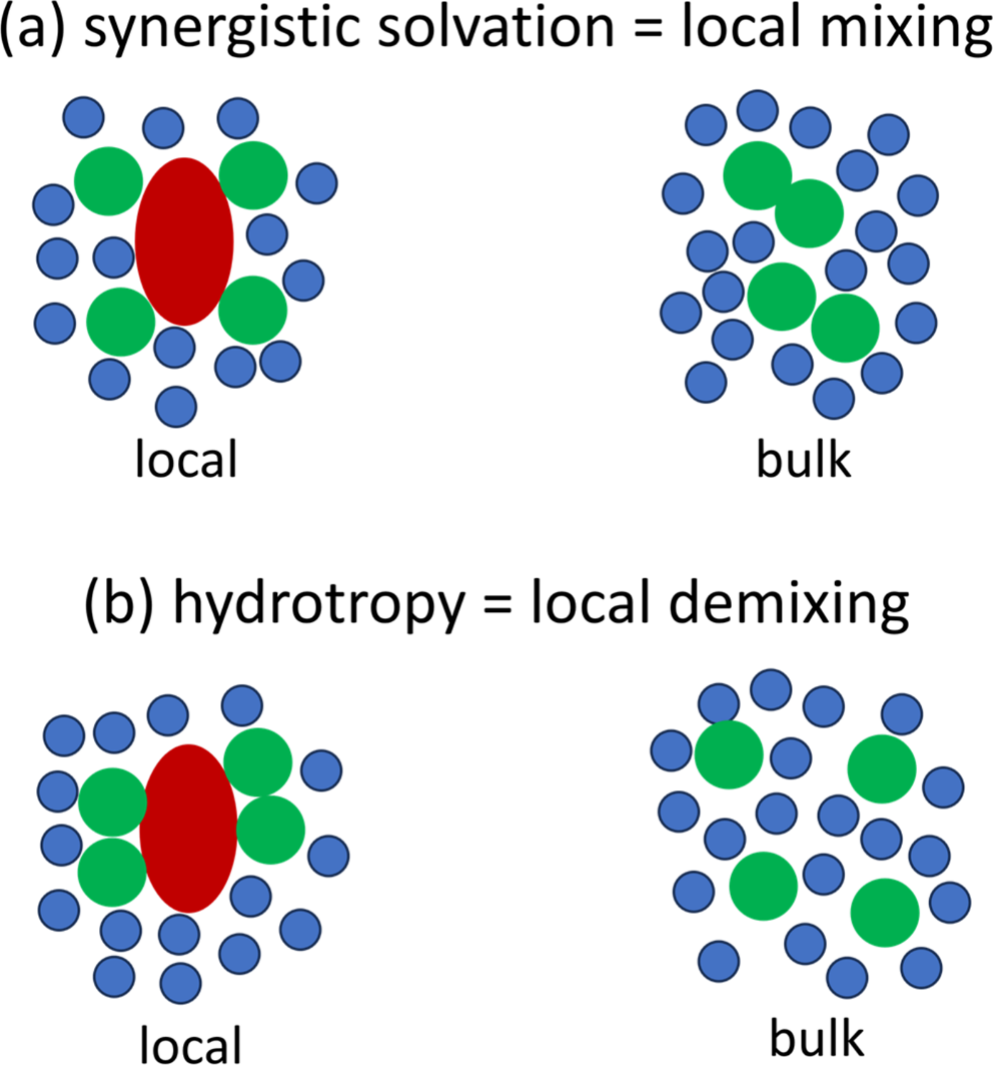
Difference between (a) synergistic solvation and (b) hydrotropy as clarified by our theory. (a) Synergistic solvation is driven by local mixing, i.e., the mixing of solvent (blue) and solubilizer (green) is enhanced around the solute (red) compared to the bulk solution. (b) Hydrotropy is driven by local demixing, i.e., the enhancement of self-association around the solute compared to the bulk solution. Synergistic solvation and hydrotropy exhibit opposite behaviors.

We have shown above that synergistic solvation takes place under preferential solute–solubilizer interaction and enhanced solvent–solubilizer mixing around the solute. The above signatures of synergistic solvation, expressed schematically via Figure 4a, are contrasted with the cooperative solubilization by hydrotropes^14,38,61^ and surfactants (Figure 4b),^15^ both of which involve enhanced solubilizer self-association around the solute. We emphasize that solubilizer self-association (in the case of hydrotropes and surfactants) is synonymous with solvent–solubilizer demixing, opposite to solvent–solubilizer mixing underlying synergistic solvation (Figure 4).

In the case of hydrotropy (surfactancy), the accumulation of hydrotropes (surfactants) around the solute leads to a further increase in the gradient of the solubility isotherm (Figures 4b and 1b,c). In contrast, solvent–solubilizer mixing weakens the effect of solubilizer accumulation (Figures 4a and 1a), leading to a decreased gradient in the solubility isotherm.

### 3.2 Choices for Interpretive Clarity in Solubility Isotherms

The solubility isotherm, founded on the fluctuation solution theory, is capable not only of drawing mechanistic insights into solubilization directly from experimental data but also of unifying solubilization phenomena that were classified previously into different subcategories. In the following, we will show how mechanistic interpretation could be facilitated by an appropriate choice in reporting experimental solubility isotherm data.

#### 3.2.1 Linear versus Logarithmic Solubility Isotherms

Solubility isotherms have traditionally been the linear representation (eq 7b), i.e., the plot of solubility itself against composition. However, the logarithmic representation (eq 7a) is superior for the following reasons. First, the logarithmic representation offers a better fitting capability of experimental isotherms (compare Figure 2b with Figure 3). Second, while the logarithmic scale involves two parameters ( and χ_0_, eq 7a) in the quadratic term, the linear scale involves three (, *A*_0_, and χ_0_, eq 7b); hence, interpreting the logarithmic scale isotherm is easier than the one in the linear scale.

#### 3.2.2 Mole-Fraction versus Molarity Solubility Scales

The superiority of the molarity scale for solubility over the mole-fraction scale was established decades ago,^52^ with its direct interpretation as the solute insertion process. This generality contrasts with the mole-fraction scale, which either requires activity coefficients or its restriction to dilute solubility.^52^ However, the mole-fraction scale is still used today for reporting solubility. Here, we add another reason as to why the mole-fraction scale adds complications. To this end, let us start with the following definition of the mole-fraction solubility in the constant *N*_1_ ensemble (to be consistent with eqs 1a and 1b)
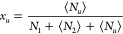
11Our goal is to derive a mole-fraction counterpart to eq 3, which is the foundation of solubility isotherms. The first step is to carry out a μ_2_-derivative of eq 11 (Appendix E), which yields
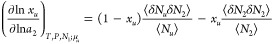
12Let us examine eq 12 as the potential generating relationship for isotherms (i.e., *x*_*u*_ as a function of *x*_2_). There are two contributions: (i)  as the measure of solute–solubilizer interaction via their number correlation and (ii)  as the bulk property. This contrasts with the molarity scale counterpart (eq 1b), which only contains (i), without any contributions from the bulk properties, (ii). Thus, the molarity scale is superior, which enables us to focus on solute–solubilizer interactions without any involvement of the bulk property.

### 3.3 Comparison to Solubility Parameters

#### 3.3.1 Solubility Parameters are Founded on the Regular Solution Theory

Synergistic solubility is conventionally explained by solubility parameters.^24^ According to this approach, solubility maximum takes place when the solubility parameter of the solute matches that of the solvent mixture, calculated as the weighted average of the parameters for the pure solvent and solubilizer.^24^ This explanation is founded on the regular solution theory.

Our objective here is to show the advantages of our new theory over the classical approach. This task is made complicated due to the gulf in foundations between the modern statistical thermodynamics of solvation (founded on the pseudochemical potential, μ_u_^*^, in eq 1a) and the classical solution thermodynamics (founded on the “ideal solubility” and the solute activity coefficient). The regular solution theory aims to provide an approximate model for the contribution of a solute’s activity coefficient to solubility.

However, in contrast to the directness afforded by modern statistical thermodynamics in achieving the aims a–c set out in Section 1.5, the regular solution model not only complicates its link to solubility via a series of assumptions but also is incapable of achieving these aims, as will be clarified in the following.

#### 3.3.2 Regular Solution Theory as the Deviation from the Ideal Solubility

The regular solution theory^21^ considers the free energy of transferring one mole of solute from the pure liquid to a regular solution
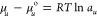
13awhere μ_u_^o^ is the chemical potential of the solute at its pure state. The regular solution theory divides this into the following two steps. The first is the transfer from the pure liquid to the ideal solution (denoted by the superscript *i*),
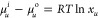
13bThe second is the transfer “from the ideal solution to a regular solution”^62^
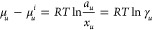
13cFor eq 13c, the regular solution theory assumes the following functional form
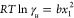
14and assumes the parameter *b* to be of enthalpic origin. The regular solution theory, in its traditional form, expresses *b* in eq 14 in terms of the “solubility parameters,” under a series of model assumptions, most notably the mixing rule, the solvent mixtures as the weighted mean of the pure solvents, and the lattice model with mean-field approximation or the van der Waals fluid model.^21^ Based on these assumptions, the solubility parameter models of Hildebrand^21^ and Hansen^24^ provide *b* via one- and three-dimensional distances between the solubility parameters of solute and solvent for the solubilities in pure solvents. For mixed solvents, their solubility parameters are estimated as the weighted mean of the constituent solvents.

What does the parameter *b* signify in eq 14? To answer this question, the following expansion from the fluctuation theory (derived by swapping the indexes 2 and *u*eq B4 in Appendix B) is useful
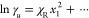
15awhere χ_R_ is expressed via the KBIs,^25^ as
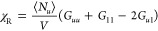
15bDue to the mathematical analogy between eqs 14 and 15a, χ_R_ in eq 15b constitutes the statistical thermodynamic interpretation for *b*/*RT* in eq 14, signifying the relative strength of the self-interactions (1 – 1 and *u* – *u*) over mutual (1 – *u*).

#### 3.3.3 Difficulties of the Solubility Parameters in Deriving Solubility Isotherms

We have rewritten the fundamental relationship for the regular solution theory (eq 14) in the language of the statistical thermodynamic fluctuation theory (eqs 15a and 15b; without adopting any of the assumptions for deriving solubility parameters). Here, we show that it is not straightforward to derive solubility isotherms from the regular solution theory (Cf. Aim b in Section 1.5). Combining eq 14 (or eqs 15a and 15b) with eq 13b and 13c, we obtain
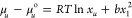
16aUnder phase equilibrium, μ_*u*_ = μ_*u*_^o^, eq 16a leads to
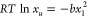
16bwhich is the solubility isotherm derived from the regular solution theory. (Noting that ln γ_*u*_ = −ln* x*_*u*_ under μ_*u*_ = μ_*u*_^o^, eq 16b for the binary mixture of the species 1 and *u* is parallel in form to eq B4 in Appendix B, expressed for the mixture of the species 1 and 2.) However, when applying eq 16b to the solvation of the solute *u* in the binary solvent mixtures (consisting of the species 1 and 2) via the solubility parameters, the following two assumptions have been introduced.^21,24^ First, *b* in eq 14 (which is used as the model approximation for χ_R_ in eq 15b) is the squared “distance” of solubility parameters between the solute and the binary mixture. Second, the solubility parameters for the binary mixture are assumed to be the composition-weighted mean of the pure (bulk) solvent values. These two assumptions have made it impossible to identify the fact that solubilization is linked to the local solution structure around the solute, which, by contrast, is captured by the fluctuation theory and its polynomial isotherm.

Thus, the solubility parameters cannot capture the microscopic basis of solvation, i.e., the local solution structure around the solute. Nevertheless, the Hansen solubility parameters have been used widely as a handy tool for solvent selection and screening.^24^ Their true foundation, applicability, and limitations should be identified via modern statistical thermodynamics.^25^ (As a preliminary step, the historic iodine dissolution experiments,^63−65^ previously taken as the supporting evidence for regular solution theory,^21^ have been reinterpreted as the dominance of enthalpy on the solvation free energy difference between solvents).^25^

### 3.4 Comparison to the Kirkwood–Buff Theory and Its Generalizations

Our quadratic isotherm marks a departure from a direct application of the KB theory^16−18^ and cooperative solubilization theory.^7,14,61,66^ Our isotherm focuses exclusively on how solubility changes with solubilizer concentration, leading to a facile identification of a few key parameters (e.g., *A*_0_ and *B*_0_, together with χ_0_) that can describe the overall functional shape of an isotherm.^43,46,55^ Such an ease contrasts with the calculation of KB integrals at every solubilizer concentration, for which measurements other than solubility (e.g., activity, density, and compressibility) are indispensable. The simplification here owes itself to the mathematical simplicity of the isotherm, which can nevertheless capture (a) solute–solubilizer interaction and (b) solute-solubilizer mixing enhancement around the solute that would require significantly more work on data processing when approached via a direct application of the KB theory.^7,14,15^ Thus, the solubility isotherm, despite its simplicity in form, captures the salient mechanisms of solubilization. However, a direct application of the KB theory will remain a powerful approach when an exhaustive quantification is needed for all interactions.

## 4 Conclusions

Our goal was twofold: (i) to elucidate the mechanism underlying synergistic solvation (i.e., the maximum solubility in binary solvent mixtures being larger than the weighted mean of the solubilities in pure solvents) and (ii) to achieve (i) via the solubility isotherm theory as a simpler alternative to the Kirkwood–Buff and the cooperative solvation theories. This was made possible by combining our recent theoretical progress: capturing solution structure and its change around the solute via molecular distribution functions and number correlations,^14,15,32,61^ a systematic approach for deriving isotherm equations directly from the principles of the fluctuation theory,^19,20,29,41,42^ and the ability to generalize our approach to isotherms to solvation via the mathematical analogy between solvation and sorption isotherms.^19,41,42^

Synergistic solvation is caused by the enhancement of solvent mixing around the solute. This behavior is opposite to hydrotropy, which involves the demixing of water and hydrotropes (i.e., self-association) around the solute. This conclusion was reached via the solubility isotherm derived from the statistical thermodynamic fluctuation theory. While the solubility isotherm provides a clear interpretation of its parameters that are rooted in the molecular distribution functions and the Kirkwood–Buff integrals, its application to experimental solubility isotherms is far less demanding than the evaluation of the Kirkwood–Buff integrals via cumbersome data analysis involving additional experimental data (e.g., density, compressibility, and osmotic data).^14,15,32,61^

Historically, the study of solubilizers has relied on classifying them into subcategories (e.g., cosolvents, hydrotropes, and surfactants) that involved challenging outliers and misleading mechanistic hypotheses. Instead, our simple, isotherm-based approach provides a universal language of solubilization.^20,43,44^

## Appendix A Statistical Variable Transformation and a Link to Solubility

First, we carry out a statistical variable transformation^48,49^ to link solubility measurements to KBIs. If approached thermodynamically, converting thermodynamic fluctuation expressed in one ensemble (e.g., {*N*_1_, μ_2_, μ_*u*_, *P*, *T*}, abbreviated as {*N*_1_} below) to another (e.g., {μ_1_, μ_2_, μ_*u*_, *V*, *T*}, abbreviated as {μ_1_}) involves a cumbersome change of variables that need to be carried out for the elements of the fluctuation Hessian matrix.^49^ In contrast, significant simplification comes from statistical variable transformation.^48,49^ The starting point is the invariance of the solubilizer–solvent mole ratio, *C*_2_ = *N*_2_/*N*_1_, and its deviation from the mean, as

A1

The Maclaurin expansion of eq A1 yields

A2

Likewise, from the invariance of *C*_*u*_ = *N*_*u*_/*N*_1_, we can also derive

A3

Equations A2 and A3 are the relationships for statistical variable transformation between {*N*_1_, μ_2_, μ_*u*_, *P*, *T*} and {μ_1_, μ_2_, μ_*u*_, *V*, *T*} that will be used throughout this paper.

Now, we use eqs A2 and A3 to carry out a {*N*_1_, μ_2_, μ_*u*_, *P*, *T*} → {μ_1_, μ_2_, μ_*u*_, *V*, *T*} transformation of eq 1a, which yields

A4

where *C*_*u*_ → 0 has been used. To simplify eq A4 further, we will introduce the Kirkwood–Buff integrals,^16,48,49^

A5

through which eqs 1a and A4 can be expressed as

A6

Note that eq A6 is a well-known relationship in the Kirkwood–Buff theory.^19,29^ When solute molecules are under phase equilibrium, a generalization of eq A6 to arbitrary solute concetrations can be carried out as shown in Appendix A of ref (15).

Second, we link the solvation free energy (pseudochemical potential), μ_*u*_^*^, to solubility. This can be carried out by decomposing the chemical potential of the solute in the solution phase, μ_*u*_, into the following two contributions: (i) the pseudochemical potential μ_*u*_^*^, which has a clear physical meaning as the free energy of inserting a solute molecule at a fixed position and (ii) the free energy of liberating a solute from positional fixation, via^52,67^

A7

where the contribution (ii), i.e., the second term of eq A7, involves ρ_*u*_ (the number density of the solute) and Λ_*u*_ (the momentum distribution function of the solute).^52,68^Equation A7 is valid for multiple-component solutions regardless of solute concentration and solvent composition.^52,68^ Differentiating eq A7 under constant temperature yields

A8

In deriving eq A8, we have exploited the fact that the only difference between ρ_*u*_ (number density) and *c*_*u*_ (molarity concentration) are their units, which leads to d ln ρ_*u*_ = d ln *c*_*u*_. When the solute in the solution phase is at phase equilibrium with the pure solute phase, μ_*u*_ = μ_*u*_^o^, where μ_*u*_^o^ is the chemical potential of the solute in its pure phase. Under constant temperature and pressure, μ_*u*_^o^ is a constant. Consequently, μ_*u*_ remains a constant, which leads to dμ_*u*_ = 0. Thus, under phase equilibrium (i.e., constant μ_*u*_),

A9

Combining eqs A6 and A9, we obtain

A10

Note that the newly appeared subscript, μ_*u*_, signifies that the partial derivative is defined under constant μ_*u*_, reflecting the derivation of eq A9. From eq A10, eq 3 can be derived straightforwardly from the elementary relationship between μ_2_ and *a*_2_.

We emphasize here that the use of the molarity (or number density) scale is advantageous because its foundation, eq A7, is “valid for any composition of the mixture.”^52^ This is in contrast to the use of the mole-fraction solubility scale whose fundamental equation has been derived “only in the limit of extremely dilute solution.”^52^

## Appendix B Interpreting the Parameters of the Polynomial Isotherm

### Activity Scale

Let us attribute to the parameters *A* and *B* a statistical thermodynamic interpretation. To facilitate the derivation, we specify the *a*_2_ → 0 limit at the end. As a first step, we rewrite *A*, defined via eq 4b, as

B1a

where *c*_1_ = ⟨*N*_1_⟩/*V* is the concentration of pure solvent. Note that  in eq B1a is reminiscent of the definition of KBI. However, the ensemble adopted for eq B1a is {*T*, *P*, *N*_1_, μ_2_} instead of the grand canonical ensemble ({*T*, *V*, μ_1_, μ_2_}) for KBIs. Consequently,  may be considered as the *pseudo*-KBI in {*T*, *P*, *N*_1_, μ_2_}, defined as

B1b

through which *A* has the following interpretation

B1c

Thus, the parameter *A* signifies solute–solubilizer interaction in the framework of the {*T*, *P*, *N*_1_, μ_2_} ensemble. In Appendix C, we have carried out a {*T*, *P*, *N*_1_, μ_2_} → {*T*, *V*, μ_1_, μ_2_} transformation, using the statistical variable transformation (Appendix A),^48,49^ and express *A* in eq B1c in the framework of the conventional KB theory.

Now, we move onto *B*, defined via eq 4c. Carrying out the differentiation, we obtain

B2a

Note that eq B2a is under the {*T*, *P*, *N*_1_, μ_2_} ensemble (instead of the grand canonical ensemble for the KB theory). Introducing the pseudo-KBI in the {*T*, *P*, *N*_1_, μ_2_} ensemble via

B2b

B2c

and noting *a*_2_ ≃ *x*_2_ ≃ ⟨*N*_2_⟩/⟨*N*_1_⟩ at *a*_2_ → 0, we obtain

B2d

for eq 4c. *B*_0_, according to eq B2d, signifies the change of solubilizer–solubilizer interaction induced by the presence of a solute molecule. This, again, is the interpretation of the {*T*, *P*, *N*_1_, μ_2_} ensemble. Transformation to the grand canonical ensemble will be carried out in Appendix C via statistical variable transformation (Appendix A).

### Conversion to the Mole-Fraction Scale

Here, we convert the variable *a*_2_ of the polynomial isotherm to the mole fraction, *x*_2_, in the form of

B3

where we have chosen the dilute-ideal standard state, which has made the coefficient for the first-order term 1. We are going to determine the parameter λ. This can be achieved by using the simplest activity model, the Margules model, which has the following form

B4

Connecting γ_1_ to *a*_2_ requires the Gibbs–Duhem equation under constant temperature and pressure

B5a

which can be rewritten as

B5b

Combining eqs B4 and B5b yields

B6

The corresponding expression from eq B3 is

B7

Comparing eqs B6 and B7 yields λ = −2α; hence, we obtain the following activity expansion using the Margules constant, α, as

B8

whose parameter will be given a statistical thermodynamic interpretation in Appendix C.^25^

Our theory is applicable when species 2 is a salt consisting of several ions. In this case, following the well-established method in the KB theory, *x*_2_ is taken as the mole fraction of total ions, *a*_2_ is the activity of the average ion, and all of the resultant KBIs refer to the interactions with the average ion.^69^ Indeed, we emphasize that salt concentrations, instead of individual ion concentrations, are controlled when changing the composition of the mixture experimentally.

### Application to Data Fitting

When we use eq 7a directly to fit the data, (*c*_*u*_)_0_, i.e., the solubility at *x*_2_ = 0, is taken as the value without any errors. To prevent this, we introduce

B9

such that the solubility at *x*_2_ = 0, ln(*c*_*u*_)_0_ + ϵ, is also a fitting parameter. Consequently, ϵ signifies the difference between experimental and fitted logarithmic solubility at *x*_2_ = 0.

## Appendix C Interpretation of the Polynomial Isotherm Parameters in the Grand Canonical Ensemble

### Ensemble Transformation: {*T*, *P*, *N*_1_, μ_2_} → {*T*, *V*, μ_1_, μ_2_}

To interpret parameters *A* and *B* in the grand canonical ({*T*, *V*, μ_1_, μ_2_}) ensemble, here we carry out a {*T*, *P*, *N*_1_, μ_2_} → {*T*, *V*, μ_1_, μ_2_} transformation via statistical variable transformation (Appendix A).

To carry out the statistical variable transformation on *A*, the inhomogeneous ensemble average must first be expressed in terms of the homogeneous,^14^ i.e.

C1a

Carrying out the statistical variable transformation on δ*N*_*u*_ and δ*N*_2_ (Appendix A), we obtain

C1b

Hence, the parameter *A*, in the grand canonical ensemble, can be rewritten as

C2

This means that *A* signifies the preferential solute–solubilizer interaction compared to solute–solvent.

To carry out statistical variable transformation on *B*, we need to convert *G*_22_^′^ and *G*_*u*,22_^′^. The key is the following statistical variable conversion (Appendix A)

C3a

Here, we introduce the conventional KBI in the grand canonical ensemble, defined as

C3b

Using eq C3b, eq C3a can be expressed as

C3c

This leads to the following transformation rule between the pseudo-KBI in {*T*, *P*, *N*_1_, μ_2_} (eq B2b) and the conventional KBI in {*T*, *V*, μ_1_, μ_2_}

C4a

This can easily be generalized as

C4b

Thus, when expressed in the grand canonical ({*T*, *V*, μ_1_, μ_2_}) ensemble, the pseudo-KBI signifies the net self-interaction, i.e., the difference between the self (solvent–solvent, *G*_11_, and solubilizer–solubilizer, *G*_22_) and mutual (solvent–solubilizer, *G*_12_) interactions. The presence of *V*/⟨*N*_1_⟩ in eq C4a (or *V*/⟨*N*_1_⟩_*u*_ in eq C4b) can be justified by considering a system in which species 2 interacts weakly with itself as well as with species 1, such that *G*_22_ ≃ 0 and *G*_12_ ≃ 0. Since *G*_11_ ≃ −*V*/⟨*N*_1_⟩ for pure solvent,^19^ this non-interacting solubilizer example leads to *G*_22_^′^ = *G*_11_ + *G*_22_ – 2*G*_12_ + *V*/⟨*N*_1_⟩ ≃ 0.

### Kirkwood–Buff χ

Following our previous papers,^43,44^ here we introduce the Kirkwood–Buff (KB) χ parameter as the measure of self-interaction as

C5a

through which eq C3c can be expressed as

C5b

Through eqs C5a and C5b, together with their inhomogeneous counterparts and the  at *a*_2_ → 0, *B*_0_ in eq B2a can be rewritten as

C6a

where *K*_e_ is the equilibrium constant for the swapping of “a solubilizer in bulk” and “a solvent in solute’s vicinity”, defined as

C6b

where *K*_1_ and *K*_2_ are the bulk-to-solute vicinity exchange constant of the solvent and solubilizer, respectively. Consequently, *B*_0_ contains the effect of solute-induced enhancement of self-interaction, but the presence of ⟨*N*_1_⟩_*u*_ and ⟨*N*_1_⟩ complicates its interpretation.

A simpler interpretation can be obtained for . Combining eq C6a with eqs B1a and C6b yields

C7

Now, eq C7 does not contain ⟨*N*_1_⟩_*u*_ and ⟨*N*_1_⟩. It signifies the enhancement of self-interactions (i.e., KB χ) around the solute from the bulk.

The Margules parameter, α, has also been given a statistical thermodynamic interpretation. Leaving the derivation to our recent papers,^25,70^ here we emphasize that α can also be expressed in terms of the bulk Kirkwood–Buff χ parameter as

C8

through which the expansion of *a*_2_ in the power series of *x*_2_ (eq B8) can be rewritten as

C9

Thus, the Kirkwood–Buff χ plays a central role in the nonlinear term of solubilization isotherms.

## Appendix D Alternative Expression for *B*/*A*

This appendix provides an alternative expression for *B*/*A* in the homogeneous ensemble. Our starting point is the fluctuation expressions of *A* and *B* (eqs B1a and B2a), from which we obtain

D1a

with

D1b

based on the definition of the mean in the inhomogeneous ensemble, ⟨*N*_2_⟩_*u*_ = ⟨*N*_2_*N*_*u*_⟩/⟨*N*_*u*_⟩. Our goal is to simplify eq D1a. The first step is to use (for derivation, see below)

D2

through which eq D1a can be rewritten with the help of eq D1b, as

D3

Combining eqs D3 and 4b, we obtain

D4

### Proof of Equation D2

From the definition of δ, the left-hand side of eq D2 can be rewritten as

D5a

Using the definition of the inhomogeneous mean

D5b

Combining eqs D5a and D5b, we obtain

D5c

The first term of the right-hand side of eq D2 becomes

D6

From eqs D5c and D6, straightforward algebra yields

D7

which, via eq D1b, is equivalent to eq D2.

## Appendix E Local Fluctuation in the Mole-Fraction Solubility Scale

Here, we demonstrate that the use of the mole-fraction solubility scale leads to a more complicated version of eq 1b. Let us start with the definition of solubility in the mole-fraction scale, written as eq 11. Here, we adopt the constant *N*_1_ ensemble (to be consistent with eq 1b). Our goal is to derive a mole-fraction version of eq 1b. The first step is to carry out a μ_2_-derivative of eq 11, which yields

E1

This leads to eq 12.
